# Supplementary material for: Human glia can both induce and rescue aspects of disease phenotype in Huntington disease
Source: Nat Commun. 2016 Jun 7;7:11758. doi: 10.1038/ncomms11758 (PMC4899632; doi:10.1038/ncomms11758)
Supplement: Supplementary Information — Supplementary Figures 1-5 and Supplementary Tables 1-9 [file ncomms11758-s1.pdf]

## SUPPLEMENTARY FIGURES

### Supplementary Figure 1 (Related to Figure 3)

### Chimerization of normal striata by Q23 and Q73 HTT-transduced hGPCs

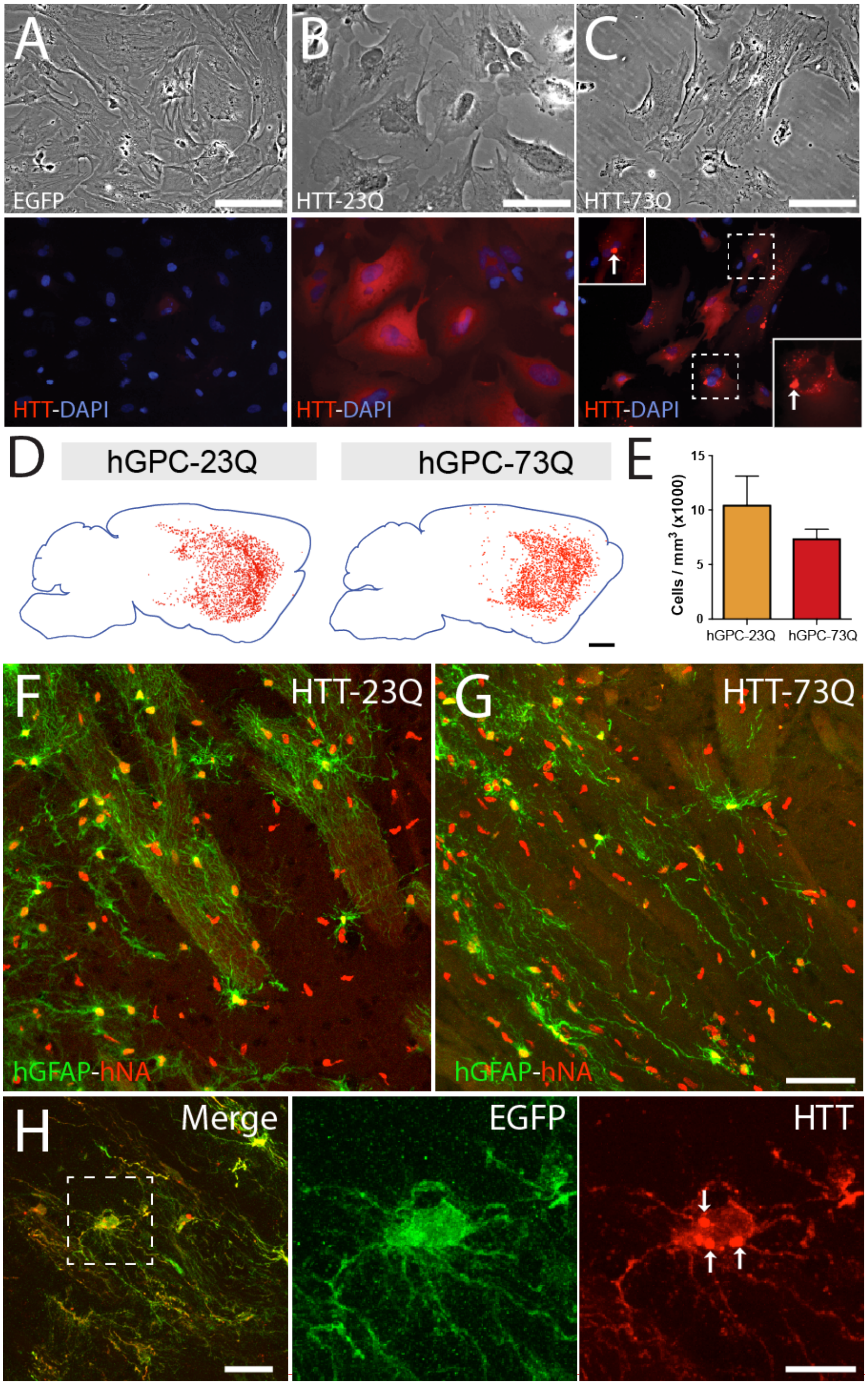


*See caption on next page.*

**Supplementary Figure 1** *(see image on previous page)*

**Chimerization of normal striata by Q23 and Q73 HTT-transduced hGPCs**

**A-C**, Glia derived from Q73 mHtt-transduced hGPCs developed inclusions. **A**, Astroglia derived from hGPCs; no Htt immunostaining was detectable in untreated cells. **B**, Q23 HTT-transduced cells (23 CAG repeats in exon 1 of HTT) overexpress cytoplasmic Htt, but no aggregates or inclusions are noted. **C**, Q73 mHtt-expressing astroglia express both high levels of Htt, and discrete cytosolic inclusions of mutant Htt protein. **D-G,** Histological analysis revealed dense engraftment by human donor cells, in both the Q23 and Q73. mHtt glial-engrafted chimeric striata, whose distributions (**D**) and densities (**E**) of human nuclear antigen $(hNA)^+$ donor cells were similar at the 12 week time-point at which electrophysio-logical recordings were obtained. The distribution of donor-derived astroglia, revealed by anti-human GFAP, was also similar in Q23 (**F**) and Q73 (**G**) chimeric brains. **H**, Donor-derived glia transduced to express Q73 mHtt (which co-express EGFP, after lenti-mHtt-EGFP transduction) develop cytosolic inclusions (*arrows*) in vivo, 12 weeks after neonatal graft. Scale: **A-C**, 50 µm; **D**, 1 mm; **F-H**, 100 µm.

**Supplementary Figure 2 (Related to Figure 3)**

**Representative recorded striatal neurons in Q23 and Q73 HTT hGPC-colonized striata**

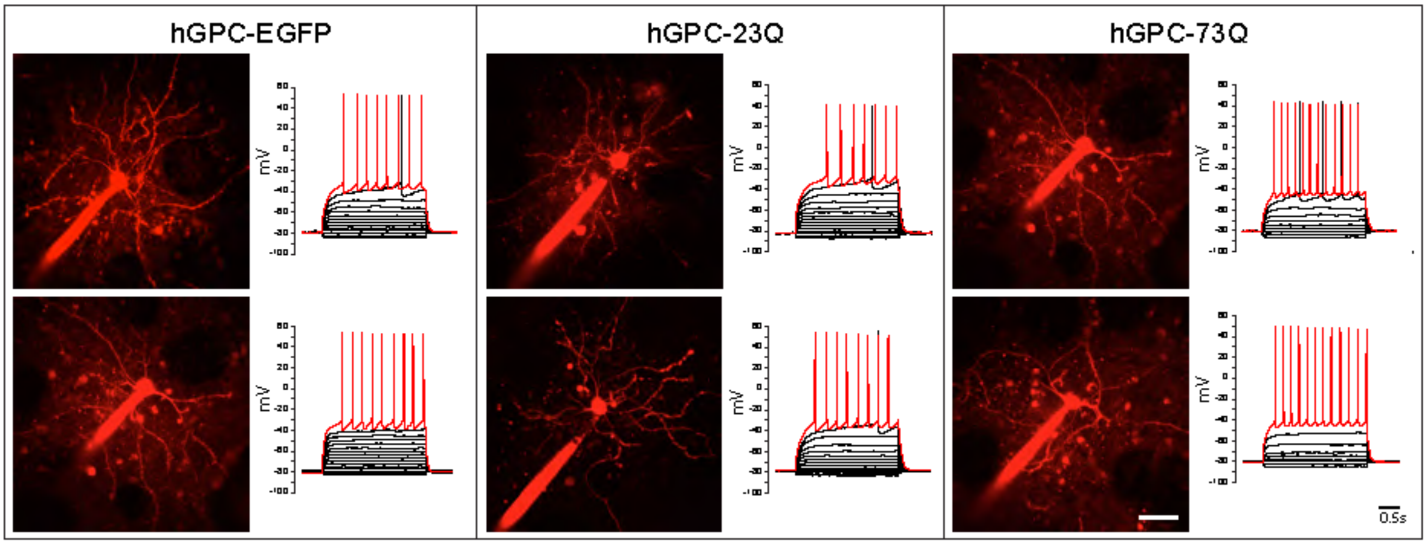


Examples of striatal neurons in hGPC-EGFP, hGPC-23Q and hGPC-73Q chimeric striata. Neurons, sampled and identified randomly throughout the striatum, were subjected to whole cell patch clamp in current-clamped configuration, and their responses to current injection recorded. The neurons displayed typical medium spiny neuron morphologies when filled with Alexa-594. Scale: 50 μm.

**Supplementary Figure 3 (Related to Figure 4)**

**Fetal human CD44$^{+}$GPCs were used to colonize the R6/2 mouse striatum**

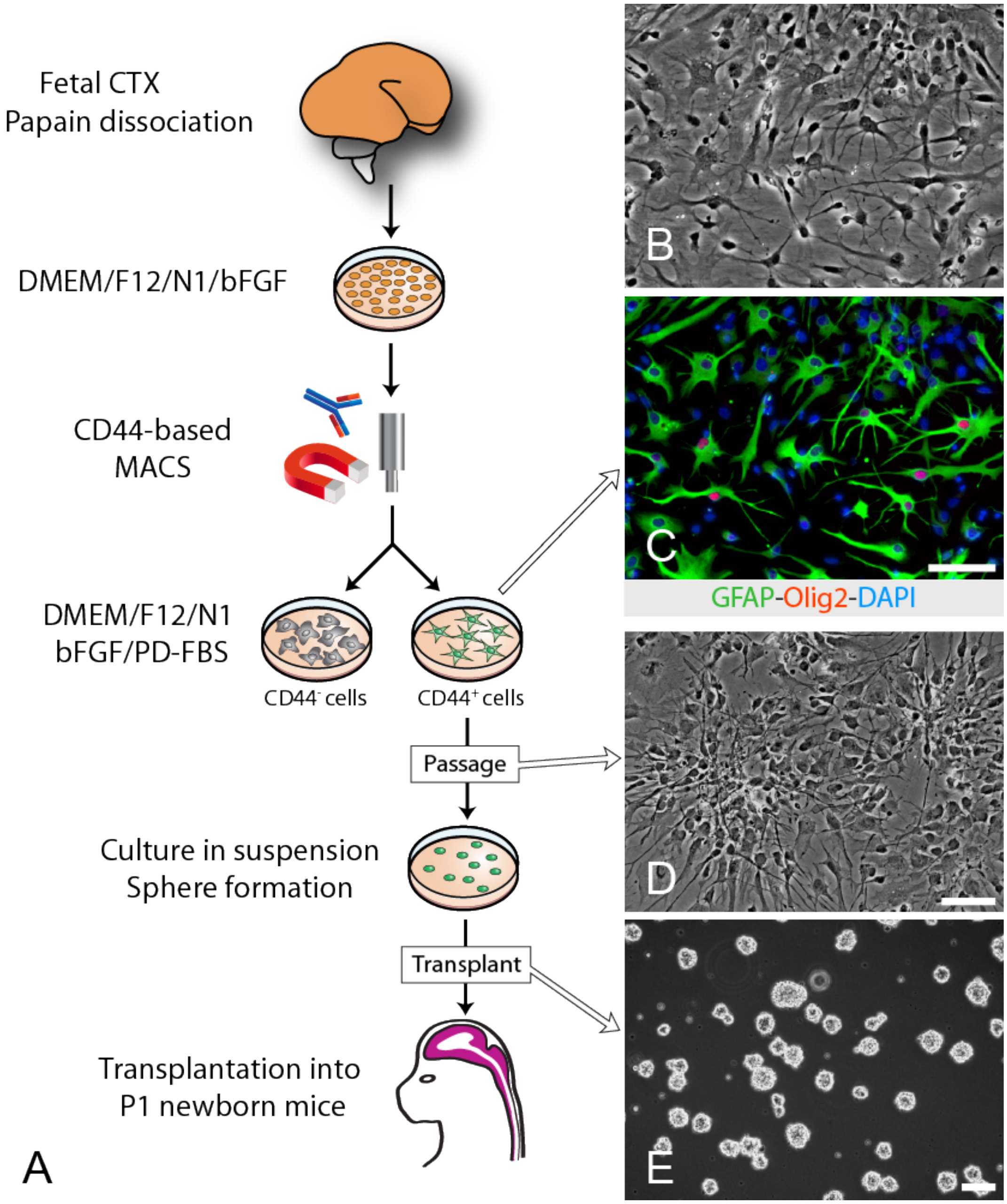


This figure exhibits the morphology and antigenicity of hGPCs derived from 18-22 wk gestational age human fetal forebrain. **A**, The schematic illustrates the major steps involved in isolating and culturing CD44-defined astrocyte-biased hGPCs, prior to their transplantation into neonatal mice. **B** shows the appearance of the CD44-sorted cells after isolation. **C**, The cells include a mix of GFAP$^{+}$/olig2$^{-}$ astrocytes and GFAP$^{+}$/olig2$^{+}$ hGPCs. **D-E**, The cells were grown to confluency and passaged (**D**), then cultured on ultralow attachment dishes for 2-5 days, in which they formed clusters of 50-100 µm diameter; these were spun and collected for transplant (**E**). Scale: 100 µm.

**Supplementary Figure 4 (Related to Figure 6)**

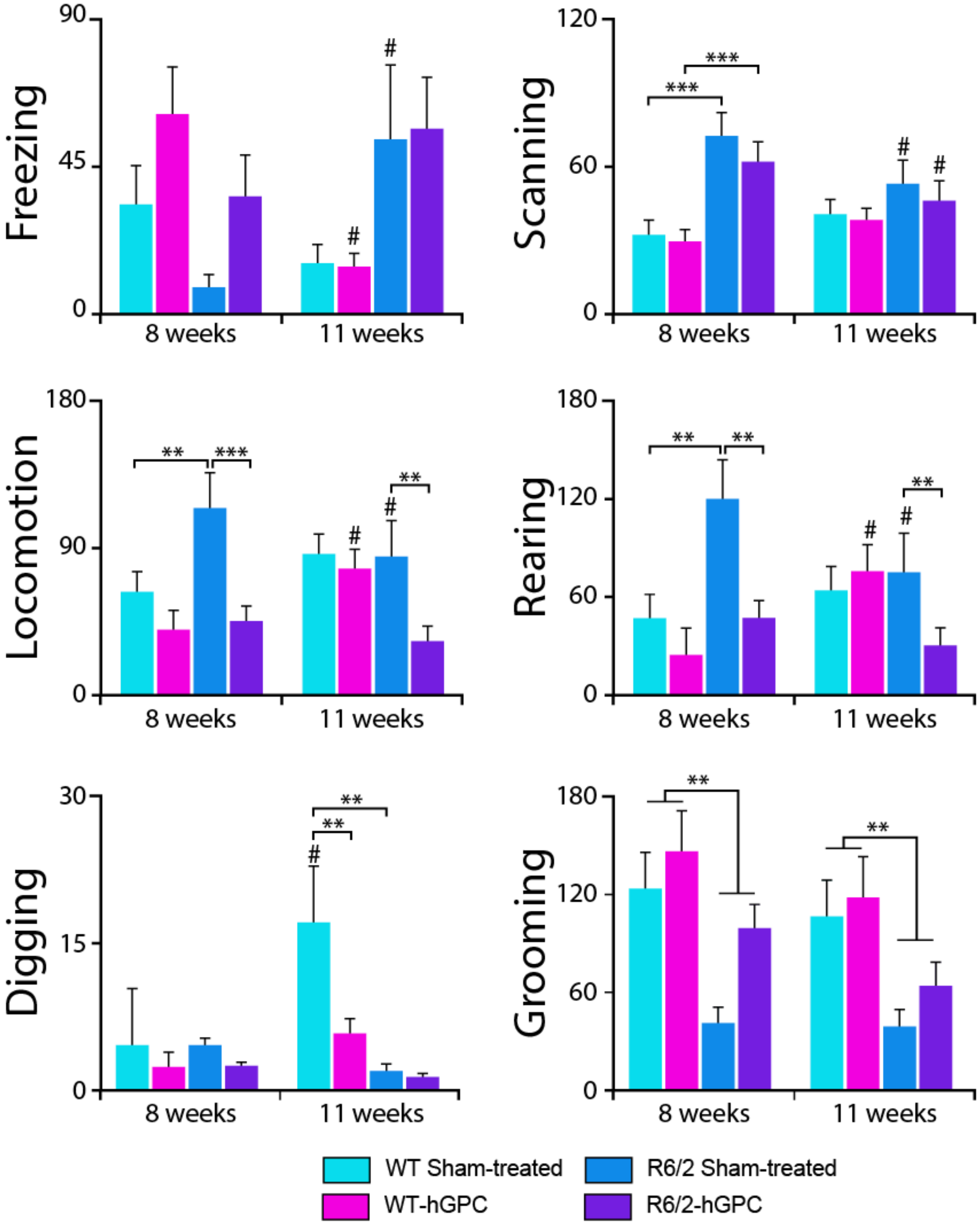


**Chimerization with normal glia partially rescued behavioral phenotype in SmartCube**

Using the SmartCube battery of behavioral tests (Psychogenics, Inc.), by 8 weeks of age sham-treated R6/2 mice showed more scanning, locomotion and rearing than wild-type controls; these disease-associated features were either attenuated or completely normalized by hGPC treatment. A reduction in grooming at both ages was marginally normalized by hGPC treatment. At 11 weeks the hyperactivity was no longer evident, freezing was increased, and digging decreased. hGPC treatment in the R6/2 mice reduced locomotion and rearing, but did not affect digging. Asterisks denote significant main effects or post hoc comparisons (*, $p < 0.05$; **, $p < 0.01$; ***, $p < 0.001$.)

**Supplementary Figure 5 (Related to Figure 6)**

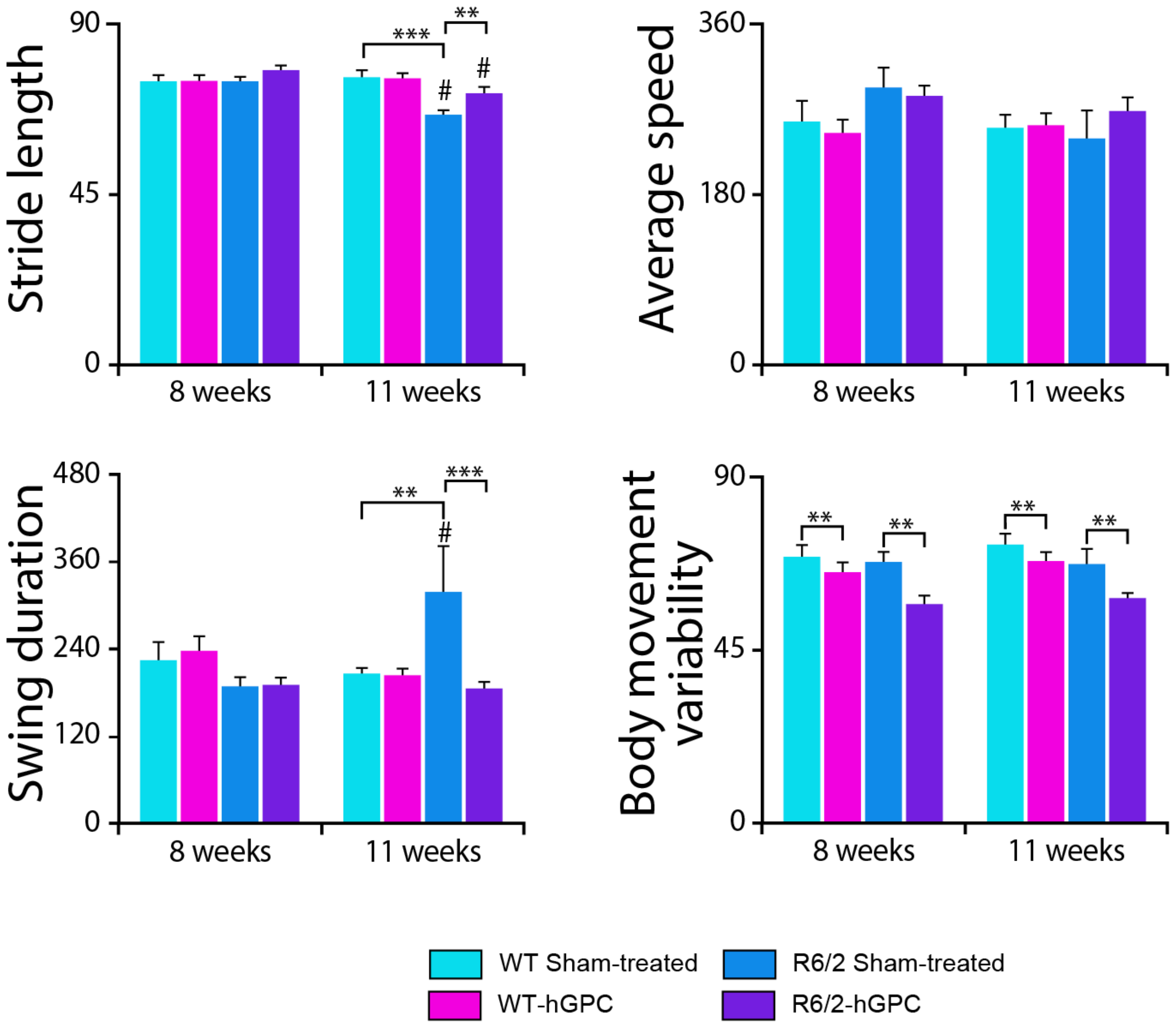


**Treatment with hGPC partially rescued disease signatures in NeuroCube**

At 8 weeks of age there were no differences in gait or related features. At 11 weeks of age deficits in gait (reduced stride length and increased swing duration) were rescued by hGPC treatment. Similarly, GPC treatment decreased body movement variability at both ages and in both the WT and R6/2 mice. Other features (e.g. speed) were not affected by genotype or treatment. Asterisks denote significant main effects or post hocs and numerals indicate age effects for a particular group; arrows indicate the effect of treatment independently of genotype and age (one symbol: $p < 0.05$; two symbols: $p < 0.01$; three symbols: $p < 0.001$.)

## SUPPLEMENTARY TABLES

### Supplementary Table 1 (Related to Methods)

Mouse allocations to each experiment

| Mouse strain | Treatment | Experimental endpoint | n |
|---|---|---|---|
| **R6/2 - Rag$^{-/-}$** | P1 mice transplanted with fetal-derived glia | Histology | 57 |
| | | Rotarod | 36 |
| | | Survival | 57 |
| | | Electrophysiology including interstitial $K^+$ measurement | 49 |
| | | Cognitive assessment | 75 |
| **Rag$^{-/-}$** | Neonatal graft of fetal-derived hGPCs expressing Q23/Q73 Htt | Histology | 16 |
| | | Electrophysiology | 36 |
| **Rag$^{-/-}$** | Neonatal graft of ESC-derived glia | Histology | 16 |
| | | Rotarod | 91 |

### Supplementary Table 2 (Related to Fig. 2)

**Rotarod performance of HD ESC-derived glial chimerized mice**

Statistical table: p values with Tukey's multiple comparisons tests

| Comparisons of treatments | p Values | | | | | | | |
|---|---|---|---|---|---|---|---|---|
| | 8 weeks | 12 weeks | 16 Weeks | 20 Weeks | 24 Weeks | 28 Weeks | 32 Weeks | 36 Weeks |
| Genea-19 (n=28) vs. Genea-20 (n=31) | 0.7966 | 0.0299 | < 0.0001 | < 0.0001 | 0.0002 | < 0.0001 | 0.0746 | 0.0565 |
| Genea-19 (n=28) vs. Untreated (n=21) | 0.9419 | 0.0772 | 0.2439 | 0.9988 | 0.9969 | 0.9999 | 0.2669 | 0.8725 |
| Genea-19 (n=28) vs. Sham treated (n=11) | 0.9552 | 0.9838 | 0.8064 | 0.3474 | 0.9793 | 0.8399 | 0.9849 | 0.7005 |
| Genea-20 (n=31) vs. Untreated (n=21) | 0.9935 | < 0.0001 | < 0.0001 | 0.0001 | < 0.0001 | < 0.0001 | < 0.0001 | 0.0035 |
| Genea-20 (n=31) vs. Sham treated (n=11) | 0.9986 | 0.0704 | < 0.0001 | 0.2308 | 0.0098 | 0.0107 | 0.0504 | 0.0034 |
| Untreated (n=21) vs. Sham treated (n=11) | 0.9999 | 0.4188 | 0.9255 | 0.4181 | 0.9393 | 0.8267 | 0.5768 | 0.976 |

Genea-19: GENEA19 (18Q)-derived hGPC chimeric Rag1$^{-/-}$.
Genea-20: mutant Htt GENEA20(48Q)-derived hGPC chimeric Rag1$^{-/-}$

**Supplementary Table 3 (Related to Fig. 3)**

**Mice chimeric for human mHTT-expressing glia exhibit alterations in MSN physiology**
Statistical table: p values with Tukey's multiple comparisons tests

**Supplementary Table 3A**
**Mutant HTT glial chimeric mouse MSNS have higher absolute resting membrane potentials**
Statistical table: p values, Tukey's multiple comparisons tests

| Comparisons of treatments | p Values |
|---|---|
| Untreated vs. hGPC-EGFP | ns |
| Untreated vs. hGPC-23Q | ns |
| Untreated vs. hGPC-73Q | < 0.0001 |
| hGPC-EGFP vs. hGPC-23Q | ns |
| hGPC-EGFP vs. hGPC-73Q | < 0.0001 |
| hGPC-23Q vs. hGPC-73Q | < 0.01 |

**Supplementary Table 3B**
**Mutant HTT glial chimeric mice have abnormally high MSN input resistance**
Statistical table: p values, Tukey's multiple comparisons tests

| Comparisons of treatments | p Values | |
|---|---|---|
| | $R_{Neg}$ | $R_{Pos}$ |
| Untreated vs. hGPC-EGFP | 0.5718 | 0.6339 |
| Untreated vs. hGPC-23Q | 0.9142 | 0.0178 |
| Untreated vs. hGPC-73Q | < 0.0001 | < 0.0001 |
| hGPC-EGFP vs. hGPC-23Q | 0.2028 | 0.0007 |
| hGPC-EGFP vs. hGPC-73Q | < 0.0001 | < 0.0001 |
| hGPC-23Q vs. hGPC-73Q | < 0.0001 | < 0.0001 |

**Supplementary Table S4 (Related to Figure 5)**

**Weights of R6/2 mice as function of treatment and time**

| Age | R6/2-hGPC | R6/2-Untreated |
|---|---|---|
| 8 weeks | 22.32 (n=12) | 20.33(n=11) |
| 16 weeks | 17.40 (n=8) | 18.58(n=9) |

**Supplementary Table 5 (Related to Figure 5)**

**Rotarod scores of glial chimeric R6/2 mice as function treatment and age**
Statistical table: p values with Tukey's multiple comparisons tests

| Comparisons of treatments | p values | | | |
|---|---|---|---|---|
| | 4 weeks | 8 weeks | 12 Weeks | 16 Weeks |
| R6/2-hGPC vs. R6/2-untreated | 0.919 | 0.937 | 0.044* | 0.558 |
| R6/2-hGPC vs. R6/2-Saline | 0.706 | 0.421 | 0.012* | 0.178 |
| R6/2-untreated vs. R6/2-Saline | 0.512 | 0.735 | 0.9 | 0.979 |

Of note, while **Supplementary Table 5** shows the data comparing treated and control mice through the 16-week time point. However, no similar analysis was possible for the 20 week time point, as all of the control animals had either died or were unable to balance on the rod by then.

**Supplementary Table 6 (Related to Figure 5)**

**Analysis of survival of hGPC-engrafted R6/2 mice by gender**

| | Male survival (d) | Female survival (d) | Statistics p value (t test) |
|---|---|---|---|
| R6/2-hGPC | 18.78 (n=16) | 19.57 (n=13) | 0.936 |
| R6/2-untreated | 18 (n=13) | 16.85 (n=15) | 0.249 |

**Supplementary Table 7 (Related to Figure 5)**

**Striatal volumes of glial chimeric R6/2 mice as a dual function of treatment and age**
Statistical table: p values with Tukey's multiple comparisons tests

| Comparisons of treatments | p values | | |
|---|---|---|---|
| | 12 weeks | 16 weeks | 20 weeks |
| WT-untreated vs. R6/2-untreated | 0.9985 | 0.0041 | < 0.0001 |
| WT-untreated vs. R6/2-hGPC | 0.8368 | 0.3447 | 0.0585 |
| R6/2-untreated vs. R6/2-hGPC | 0.8436 | 0.1224 | 0.0066 |

WT: $Rag1^{-/-}$ immunodeficient mice; R6/2: $Rag1^{-/-}$ x R6/2(120CAG).

**Supplementary Table 8 (Related to Fig. 7)**

**Human glial chimerized R6/2 mice exhibit restoration of MSN physiological parameters**
Statistical table: p values with Tukey's multiple comparisons tests. All in WT: Rag1$^{-/-}$ immunodeficient mice.

**A. Input resistance (Related to Fig. 7)**

| Comparisons of treatments | p Values | |
|---|---|---|
| | $R_{Neg}$ | $R_{Pos}$ |
| WT-untreated vs. R6/2-untreated | 2.87439E-8 | 2.02057E-20 |
| WT-untreated vs. R6/2-hGPC | 6.67309E-5 | 1.01578E-12 |
| R6/2-untreated vs. R6/2-hGPC | 0.8760 | 0.0073 |
| WT-untreated vs. WT-hGPC | 0.5048 | 0.9899 |

**B. EPSC Frequencies (Related to Fig. 7E)**

| Comparisons of treatments | p Values | |
|---|---|---|
| | sEPSC | mEPSC |
| WT-untreated vs. R6/2-untreated | 6.88787E-7 | 0.1879 |
| WT-untreated vs. R6/2-hGPC | 0.0126 | 0.4888 |
| R6/2-untreated vs. R6/2-hGPC | 0.0077 | 0.1333 |
| WT-untreated vs. WT-hGPC | 0.0339 | 0.4814 |

**C. EPSC Amplitudes (Related to Fig. 7F)**

| Comparisons of treatments | p Values | |
|---|---|---|
| | sEPSC | mEPSC |
| WT-untreated vs. R6/2-untreated | 0.1178 | 0.5478 |
| WT-untreated vs. R6/2-hGPC | 0.7011 | 0.6753 |
| R6/2-untreated vs. R6/2-hGPC | 0.2054 | 0.7990 |
| WT-untreated vs. WT-hGPC | 0.0443 | 0.9728 |

**Supplementary Table 9 (Related to Fig. 8)**

**Striatal interstitial potassium levels**
Statistical table: p values with Tukey's multiple comparisons tests

| Comparisons of treatments | p Values |
|---|---|
| WT-untreated vs. WT–hGPC | 0.3701 |
| WT-untreated vs. R6/2-untreated | < 0.0001 |
| WT-untreated vs. R6/2-hGPC | 0.3163 |
| WT-hGPC vs. R6/2-untreated | 0.0023 |
| WT hGPC vs. R6/2-hGPC | 0.9999 |
| R6/2-untreated vs. R6/2-hGPC | 0.0007 |

WT: Rag1$^{-/-}$ immunodeficient mice; R6/2: Rag1$^{-/-}$ x R6/2(120CAG).
